# Geographical Variation in the Gut Microbiota of *Duttaphrynus melanostictus* : Relative Contributions of Environmental Filtering and Host Physiological Modulation

**DOI:** 10.1002/ece3.73993

**Published:** 2026-07-09

**Authors:** Di K. Zhu, Nan Wen, Ke Y. Peng, Lin L. Jiang, Jin Li, Li Zhao

**Affiliations:** ^1^ Key Laboratory of Southwest China Wildlife Resources Conservation (Ministry of Education) China West Normal University Nanchong China; ^2^ Key Laboratory of Artificial Propagation and Utilization in Anurans of Nanchong City China West Normal University Nanchong China; ^3^ Key Laboratory of National Forestry and Grassland Administration on Biodiversity Conservation on the Qinghai‐Xizang Plateau China West Normal University Nanchong China

**Keywords:** *Duttaphrynus melanostictus*, environmental filtering, geographical variation, gut microbiota, host physiology

## Abstract

The gut microbiota of ectothermic vertebrates often varies substantially across space, but how much of this variation stems from environmental filtering versus host physiology remains unclear. We examined gut microbial communities in 385 Asian common toads ( *Duttaphrynus melanostictus* ) from 14 populations across southern China, using 16S rRNA sequencing to assess the roles of climate, topography, and host traits in shaping community structure. Overall microbial composition stayed largely stable in adults aged 2–6 years, though secondary phyla like Actinobacteria showed gradual age‐related shifts. Abiotic factors—particularly water conductivity (a proxy for salinity/ion content), annual mean temperature, and elevation—emerged as the primary drivers. Firmicutes and Verrucomicrobiota increased with elevation, while Fusobacteriota declined. Variance partitioning confirmed that climatic and environmental variables explained most of the observed variation; host age and sex contributed only minor fine‐tuning. These results support an environment‐dominated, host‐modulated assembly model in widespread amphibians, where large‐scale filtering sets the geographical template and adult traits make secondary adjustments. This study provides a quantitative framework for understanding how amphibian gut microbiota adapt to heterogeneous environments.

## Graphical abstract

We examined gut microbial communities in 385 Asian common toads ( *Duttaphrynus melanostictus* ) from 14 populations across southern China, using 16S rRNA sequencing to assess the roles of climate, topography, and host traits in shaping community structure. Our study provides a quantitative framework for understanding how amphibian gut microbiota adapt to heterogeneous environments.

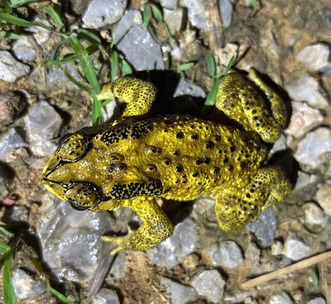

## 1 Introduction

Coevolution has forged intricate partnerships between vertebrates and their gut microbiota, which are now recognized as fundamental to host physiology (Shapira 2016; Cao et al. 2024; Park and Do 2024b). These holobiontic associations integrate host and microbial genomes into cohesive functional units shaped by evolutionary pressures (Rosenberg and Zilber‐Rosenberg 2018; Groen et al. 2018). Within the intestinal lumen, bacteria and other microbes interact with enterocytes, a molecular dialog critical for barrier integrity, metabolic balance, and immune homeostasis (Artis 2008). Functioning as an auxiliary metabolic organ, the microbiome transduces environmental cues into physiological responses, extending its influence far beyond digestion.

Microbial assembly reflects both endogenous factors such as genetics, age, and behavior and exogenous determinants including diet, drugs, and environmental contaminants (Kohl et al. 2013; Yang et al. 2025; Huang and Liao 2021; Lu et al. 2024; Zhao et al. 2024; Costello et al. 2012; Liu et al. 2025; Zhu and Xu 2025; Groombridge 1992). Yet research remains heavily skewed toward endothermic vertebrates, leaving ectotherms critically understudied (Tong et al. 2020). Among these, amphibians, particularly frogs, offer unique insights into host–microbe dynamics due to their biphasic life cycles, permeable skin, and environmental sensitivity, which together create natural experiments in microbial colonization and resilience (Knutie et al. 2018; Hof et al. 2011). Amphibians from geographically distinct populations experience heterogeneous climates, terrains, and food resources. Their intestinal microbiota may assist in coping with local environmental stress through adaptive shifts in species composition and functional pathways (Zhu, Chang, et al. 2025; Williams et al. 2023). Thus, analyzing the geographic differentiation and drivers of amphibian gut microbiota can reveal co‐adaptive laws governing host–microbe evolution (Torres‐Sánchez and Longo 2022), while also informing conservation strategies.

**FIGURE 1:**
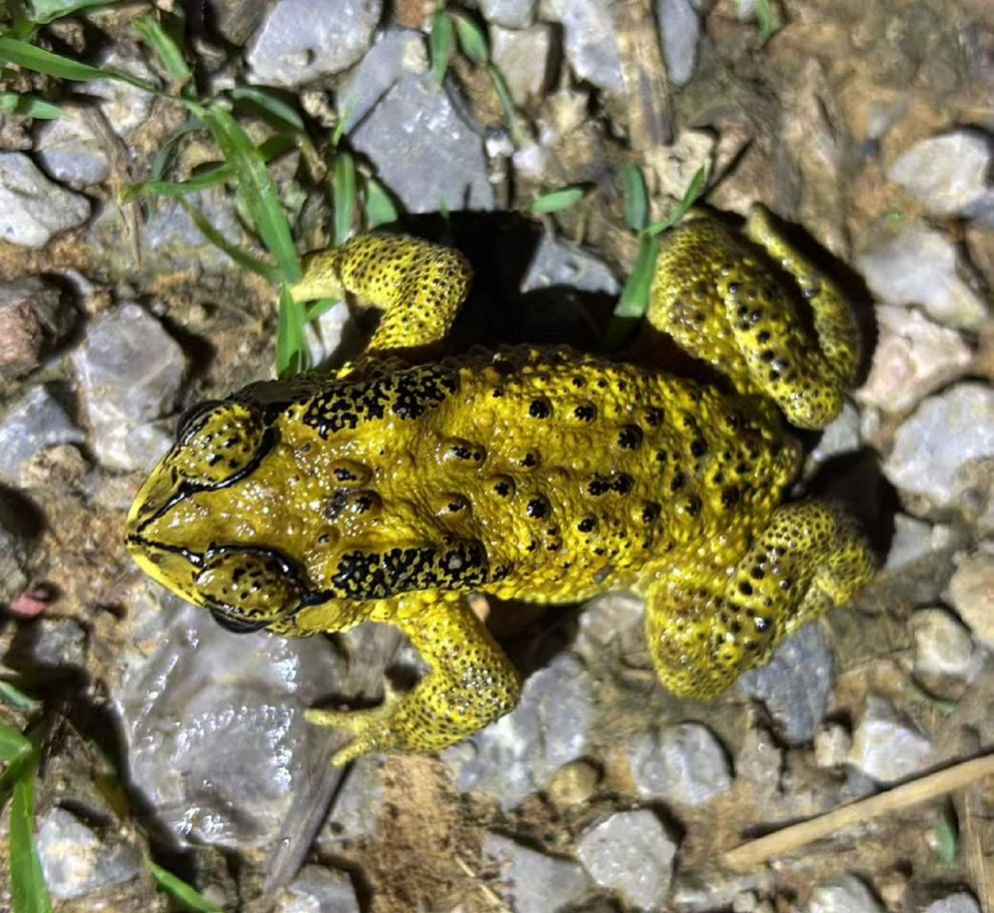
A male *D. melanostictus* . Photograph by the authors.

*Duttaphrynus melanostictus* is a highly adaptable amphibian widely distributed in southern China, serving as an excellent model for investigating host–microorganism geographical patterns (Kueneman et al. 2014; Figure 1). Despite its ecological significance, research on its gut microbiota remains nascent, focusing primarily on artificial breeding or single populations (Trevelline et al. 2019; Zhu et al. 2024). Comprehensive cross‐regional systematic studies are lacking (Zhu, Chang, et al. 2025). Existing studies emphasize individual environmental factors (e.g., temperature, pH) but lack integrated analyses quantifying the relative influences of climate, geography, and host traits (Park and Do 2024a). Moreover, age is often treated categorically (larvae vs. adults), failing to capture its continuous regulatory effect on microbial responses (Wang, He, et al. 2025; Wang, Su, et al. 2026; Shi et al. 2023). Furthermore, most analyses focus on species composition, while functional pathway adjustments to local conditions remain underexplored (Park and Do 2024a).

To address these gaps, we expanded the sample size to 385 individuals, providing a solid foundation for statistical analysis. Age was treated as a continuous variable to precisely assess its modulation of microbial responses. Climatic, environmental (including water quality), and host attributes were integrated into a unified analytical framework, using redundancy analysis, random forests, and variance partitioning to disentangle their relative contributions. We also employed PICRUSt1 to predict metabolic pathways, aiming to determine whether environmental gradients drive functional shifts in microbial communities.

Based on the above approach, we hypothesize that the geographical variation of gut microbiota in widely distributed amphibians follows an “environment‐dominated, host‐modulated” hierarchical model, where water quality (especially conductivity), annual mean temperature, and elevation play the primary role, while host age and sex provide secondary fine‐tuning. This study aims to develop such a framework and to offer a predictive basis for understanding microbiome responses to climate change and anthropogenic environmental variation.

## 2 Materials and Methods

### 2.1 Sample Collection and Age Determination

**FIGURE 2:**
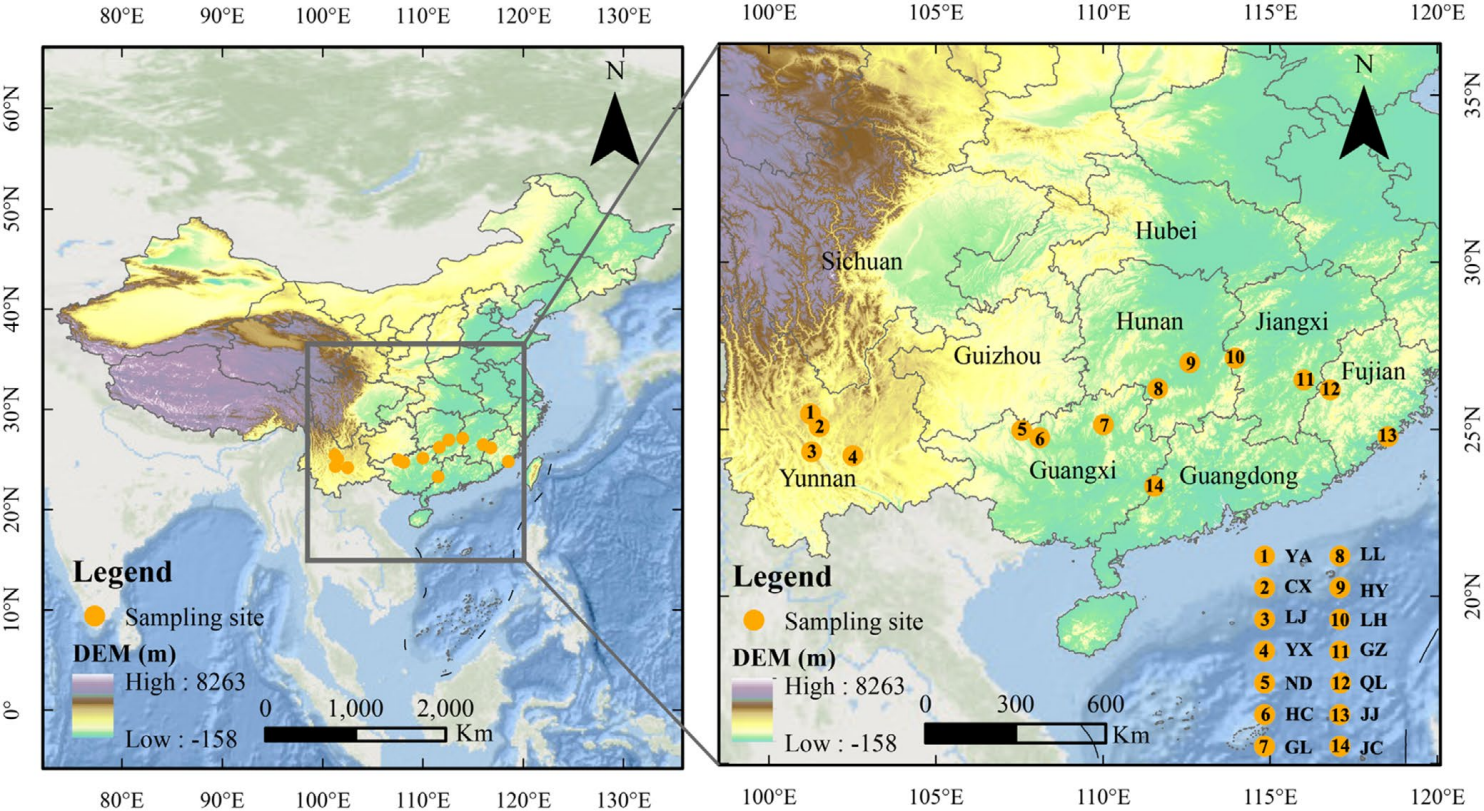
Fourteen sampling points on the *D. melanostictus* . The numbers in the figure represent sampling sites, and the detailed information of the sampling sites is shown in Table 1.

A total of 385 adult Asian common toads ( *D. melanostictus* ) individuals were collected from 14 geographical populations across southern China (including Guangdong, Yunnan, Fujian, Jiangxi, and Hunan Provinces, and Guangxi Zhuang Autonomous Region) between March and May 2025 (Figure 2; Table 1). All specimens were captured during the peak nocturnal activity period (19:00–22:00). Immediately after capture, toads were euthanized by single pithing, a method approved for amphibians. Specifically, a sharp needle was inserted through the foramen magnum to destroy the brain (cranial pithing); spinal pithing was not performed. Complete cessation of opercular (throat) movement and loss of corneal reflex were confirmed before proceeding with dissection. Dissections were carried out on the same night; intestinal contents were extracted and stored in sterile centrifuge tubes at −20°C for subsequent DNA extraction. The fourth toe of the right hind limb was amputated and fixed in 4% formaldehyde for age determination.

**TABLE 1:** Sampling sites and population characteristics of *D. melanostictus* .

Sampling points	Elevation (m)	Latitude (°N)	Longitude (°E)	Maximum age (years)	Minimum age (years)	Mean age ± SD	Males (*n*)	Females (*n*)
YA	1911	25.46220434	101.25636085	6	3	4.15 ± 1.00	28	12
CX	1783	25.08342269	101.51473900	6	3	4.83 ± 1.17	3	3
LJ	1761	24.32863929	101.28542710	6	3	4.67 ± 1.51	3	3
YX	1269	24.21077144	102.51691012	5	3	3.35 ± 0.53	40	0
ND	686	24.97482860	107.56804330	5	3	3.71 ± 0.74	30	1
HC	219	24.75111202	108.10725788	5	2	3.22 ± 0.58	21	6
GL	165	25.13754273	110.01522143	5	3	3.23 ± 0.50	28	2
LL	101	26.21851412	111.63506387	6	3	3.93 ± 0.87	8	22
HY	94	26.98860080	112.59661619	5	2	3.27 ± 0.64	21	9
LH	164	27.12376172	113.95580700	4	2	3.12 ± 0.56	15	16
GZ	168	26.48329991	116.01381443	4	3	3.25 ± 0.44	15	5
QL	347	26.18538922	116.80744479	4	2	3.17 ± 0.46	12	18
JJ	34	24.79233986	118.52379392	5	3	3.60 ± 0.67	24	6
JC	11	23.29710016	111.54452977	6	3	3.92 ± 0.81	19	15

Age was determined using the skeletochronological method (Smirina 1994). The third phalanx of the fourth toe was decalcified in 5% nitric acid (Ren's solution) for 24–72 h until transparent, then rinsed, stained with hematoxylin and eosin, and embedded in paraffin. Cross‐sections (10–12 μm) were examined under a light microscope (20×). Growth arrest lines (LAGs) were counted independently by two observers; each LAG corresponded to one winter. Age was treated as a continuous variable in all statistical analyses (McCreary et al. 2008).

### 2.2 Measurement of Local Environmental Variables

At each sampling site, three points spaced 3–5 m apart were selected for in situ measurements. At each point, three replicate readings were taken and averaged. Water conductivity (μS/cm) and total dissolved solids (TDS, ppm) were measured using a SWEVY SW‐302 m at a depth of 3–5 cm. Water pH was measured with a SWEVY SW738 meter (also at 3–5 cm depth). Dissolved oxygen (DO, mg/L) was measured using a SMART SENSOR AR8406 dissolved oxygen meter at a depth of 20–30 cm (representing the mid‐water column in shallow habitats). All meters were calibrated according to the manufacturer's instructions before each sampling session. The three point averages were used as site‐level values.

Climate data were extracted from WorldClim version 2.1 (Fick and Hijmans 2017) at 30‐arc‐second resolution. Nineteen bioclimatic variables were downloaded for each site. After removing highly correlated variables (|*r*| > 0.8), four variables were retained: annual mean temperature (bio1), temperature seasonality (bio4), temperature annual range (bio7), and annual precipitation (bio12). Elevation (m) was obtained from the SRTM digital elevation model.

### 2.3 DNA Extraction, 16S rRNA Gene Amplification and Sequencing

Genomic DNA was extracted following the protocols provided by each sample's DNA extraction kit. The quality of the extracted DNA was assessed using 1% agarose gel electrophoresis and a NanoDrop. The V3‐V4 region of the 16S rRNA gene was amplified with universal primers containing barcodes (515F/806R) and Premix Taq (Caporaso et al. 2011). The resulting PCR products were subsequently purified using the E.Z.N.A. Gel Extraction Kit. Sequencing libraries were prepared with the NEBNext Ultra II DNA Library Prep Kit for Illumina, and paired‐end sequencing was conducted on the Illumina NovaSeq 6000 platform. The original data were stored in FASTQ format (Cock et al. 2010).

### 2.4 Sequence Processing, ASV Clustering and Taxonomic Annotation

Raw reads were quality‐filtered using Fastp v.0.20.0 (Chen et al. 2018) with a sliding window of 5 bp and a minimum average quality score of Q20. Paired‐end reads were merged using FLASH v.1.2.11 (Magoč and Salzberg 2011) with a minimum overlap of 10 bp. ASV clustering was executed through methods such as Usearch (Edgar 2013), DADA2 (Callahan et al. 2016), and Deblur (Amir et al. 2017). The merged reads were denoised using the DADA2 pipeline implemented in QIIME2 (version 2024.5) to infer amplicon sequence variants (ASVs). ASVs with an abundance < 5 across all samples were discarded. Taxonomic assignment was performed using the SILVA database (version 138.1; Quast et al. 2012), Greengenes (DeSantis et al. 2006), and RDP (Cole et al. 2014) with a confidence threshold of 0.8. The resulting ASV table (samples × ASVs) was rarefied to the minimum sequencing depth (41,171 reads per sample) for subsequent diversity analyses.

### 2.5 Diversity and Community Analyses

Alpha diversity (Shannon, Chao1 and Simpson indices) was calculated using the diversity function in the R package vegan (v2.6–8). Beta diversity was assessed by Bray–Curtis dissimilarity and visualized using principal coordinate analysis (PCoA). Permutational multivariate analysis of variance (PERMANOVA, adonis2 function in vegan, 999 permutations) was used to test the effects of age, sex, sampling site, pH, conductivity, DO, elevation, and annual mean temperature (bio1) on microbial community composition (Anderson 2001).

### 2.6 Functional Prediction

Functional potential of the gut microbiota was predicted using PICRUSt1 (Langille et al. 2013) based on the ASV table and the KEGG database (Kanehisa and Goto 2000). Predicted KEGG orthologue (KO) abundances were collapsed to pathway level 3. Differentially abundant pathways between age groups or environmental gradients were identified using Spearman correlation (|*r*| > 0.6, *p* < 0.05) after Benjamini–Hochberg correction.

### 2.7 Statistical Analyses of Driving Factors

All statistical analyses were performed in R v4.5.2 (R Core Team 2025) with the packages vegan, randomForest (v4.7–1.2), and readxl (v1.4.3). Random seeds were set as 123 for permutation‐based tests and 456 for random forest.

Redundancy analysis (RDA) was performed on Hellinger‐transformed ASV data (decostand, method = “hellinger”) using eight explanatory variables: bio1, bio4, bio7, bio12, elevation, pH, conductivity, and DO (Ter Braak 1986). The significance of each variable was assessed by 999 permutations.

Variance partitioning analysis (VPA) was used to quantify the independent and joint contributions of three variable groups: climate (bio1, bio4, bio7, bio12), topography (elevation), and water quality (ph, conductivity, DO). The analysis was conducted using the varpart function in vegan on Hellingertransformed ASV data; adjusted *R* ^2^ values are reported.

Random forest regression was applied to predict Shannon diversity using all environmental and host variables (age, sex, bio1, bio4, bio7, bio12, elevation, pH, conductivity, DO) as predictors (Liaw and Wiener 2002; Breiman 2001). The randomForest function was run with ntree = 500 and mtry = 3. Variable importance was measured as the percentage increase in mean squared error (%IncMSE) when the variable was permuted.

Mantel test (mantel function in vegan, 9999 permutations) was used to evaluate the correlation between Bray–Curtis dissimilarity of gut microbiota and geographic distance (Mantel 1967).

All plots were generated with ggplot2 (v3.5.1) and the scales package.

## 3 Results

### 3.1 Horizontal Distribution of Dominant Taxa

In this study, double‐ended sequencing of the V3‐V4 region of the 16S rRNA gene was conducted on 385 intestinal content samples from *D. melanostictus* . A total of 44,106,839 original reads were generated, yielding an average of 114,564 ± 16,259 reads per sample (range 52,828–131,561). After quality control (Fastp, average Q20 = 98.0%, Q30 = 93.8%, GC content = 51.6%), 44,053,557 clean reads were retained (data utilization rate 99.88%). The number of reads per sample ranged from 41,171 to 106,844, with an average of 77,922 ± 14,856. Coverage for all 385 samples was ≥ 0.99.

Following denoising (Usearch), 188,344 ASVs were identified, with each sample containing 282–3001 ASVs (average 949 ± 463). With an average of 77,922 reads per sample after quality control, the sequencing depth was sufficient to capture the majority of microbial diversity. Phylum‐level annotation revealed that the dominant groups were Proteobacteria, Firmicutes, Fusobacteriota, and Verrucomicrobiota, with Bacteroidota and Actinobacteriota as secondary groups. Proteobacteria and Firmicutes together formed the core framework of the intestinal microbiota.

### 3.2 Gut Microbial Characteristics of the Five Age Groups

**FIGURE 3:**
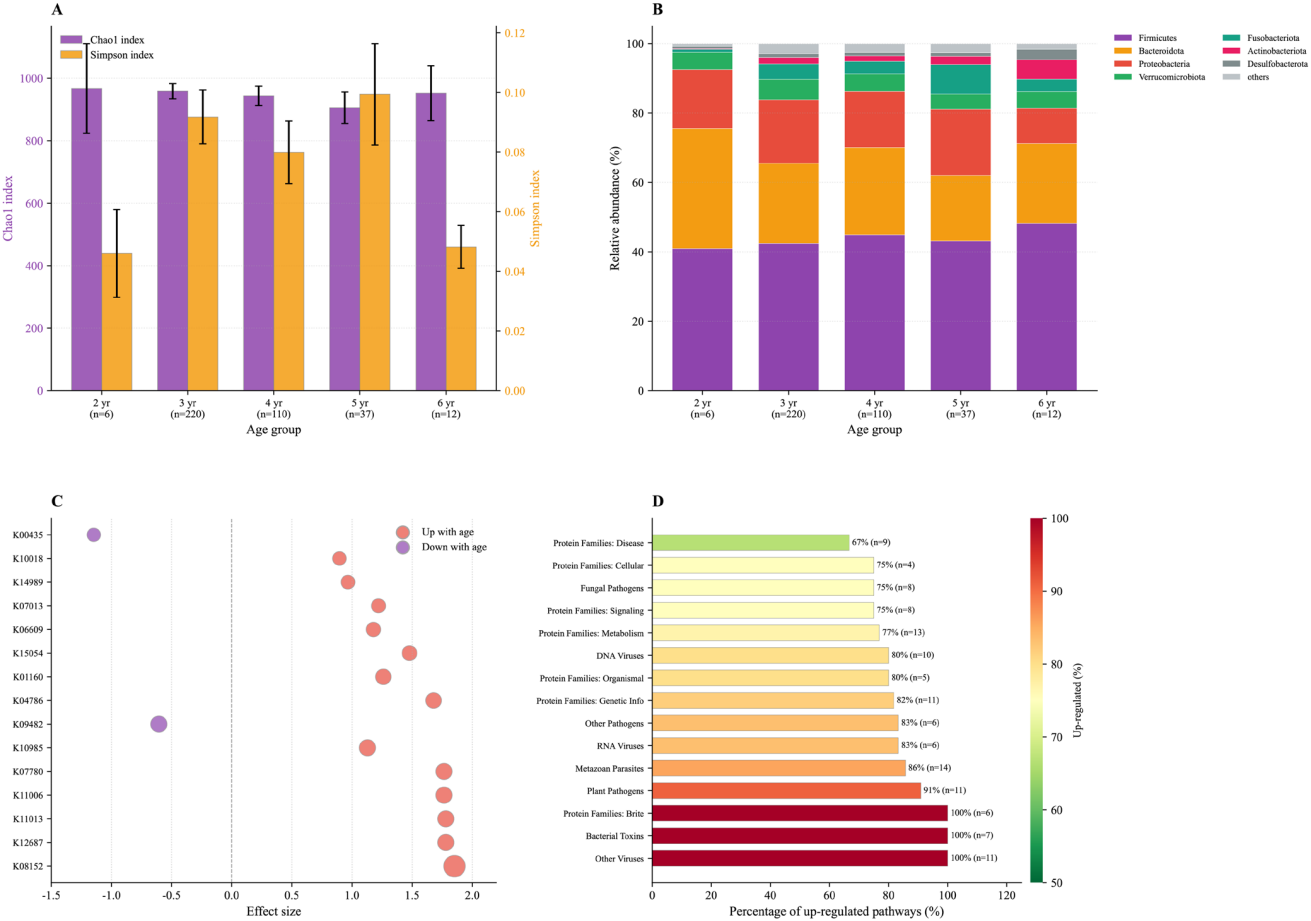
Age‐related changes in gut microbiota. (A) Alpha diversity indices (Chao1 and Simpson) across age groups. Data are presented as mean ± SEM Sample sizes: 2 years (*n* = 6), 3 years (*n* = 220), 4 years (*n* = 110), 5 years (*n* = 37), 6 years (*n* = 12). (B) Phylum‐level community composition across age groups. (C) Top 15 age‐related KEGG pathways. Bubble size represents −log_10_(*p*‐value). Red indicates upregulation with age, purple indicates downregulation. (D) Functional category distribution. Color gradient represents the percentage of upregulated pathways.

The intestinal microbiota of 2‐ to 6‐year‐old *D. melanostictus* remained largely stable while showing localized variation. Chao1 index showed no significant differences across age groups (Figure 3A). Simpson index followed a U‐shaped trajectory: relatively low in 2‐year‐olds, rising to a plateau between ages 3 and 5, then declining again in 6‐year‐olds to levels comparable to the youngest group (Figure 3A). Beta diversity PERMANOVA (Bray‐Curtis) showed no significant effect of age, and PCoA ordination revealed broad overlap among age groups.

Phylum‐level relative abundances changed with age (Figure 3B). Firmicutes increased from 40.95% (age 2) to 48.16% (age 6). Bacteroidota decreased from 34.59% (age 2) to 18.91% (age 5) before rebounding to 23.05% (age 6). Proteobacteria remained stable (16%–19%) from ages 2 to 5, then decreased to 10.14% at age 6. Actinobacteria increased linearly from 0.24% (age 2) to 5.57% (age 6), a 23‐fold increase.

Functional predictions (PICRUSt1) showed that core KEGG level‐1 pathways (carbohydrate, amino acid, energy, and lipid metabolism) did not differ significantly among age groups. At the orthologue level, however, several KEGG orthologues shifted with age (Figure 3C). Gene families associated with viral functions and bacterial toxins increased with age (e.g., K08152: 1.67 to 6.90, effect size 1.85), while genes related to cellular processes decreased. The distribution of upregulated and downregulated functional categories across age groups is summarized (Figure 3D).

### 3.3 Regulatory Effects of Climate Factors on the Structure of Gut Microbial Communities

**FIGURE 4:**
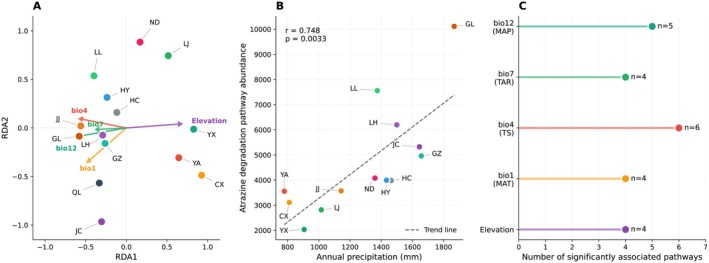
Effects of climatic factors on gut microbiota and associated functional pathways. (A) RDA biplot of 14 populations (colored circles) and five climatic factors (arrows): Annual mean temperature (bio1), temperature seasonality (bio4), temperature annual range (bio7), annual precipitation (bio12), and elevation. (B) Correlation between atrazine degradation pathway abundance (KEGG L3) and annual precipitation. (C) Number of KEGG L3 pathways significantly correlated (Spearman |*r*| > 0.6, *p* < 0.05) with each climatic factor. MAP, Annual precipitation; MAT, Mean annual temperature; TAR, Temperature annual range; TS, Temperature seasonality.

Redundancy analysis (RDA) showed that five climatic factors together explained 39.2% of the variation in gut microbial community structure (Figure 4A). Elevation had the highest absolute loading on the first RDA axis, followed by annual precipitation and temperature seasonality.

KEGG level‐3 functional annotation identified 24 pathways significantly associated with climatic factors (Spearman |*r*| > 0.6, *p* < 0.05; Figure 4B,C). The atrazine degradation pathway showed the strongest correlation with annual precipitation (Figure 4B; *r* = 0.748, *p* < 0.05). The siderophore biosynthesis pathway was negatively correlated with elevation and positively correlated with annual precipitation.

Based on these correlations, three climate‐adaptation functional modules were distinguished. The precipitation‐adaptation module (five pathways, mean |*r*| = 0.663) included atrazine degradation and bacterial invasion of epithelial cells. The temperature‐adaptation module (six pathways, mean |*r*| = 0.625) included development/regeneration and African trypanosomiasis. The elevation‐adaptation module (four pathways) showed bidirectional responses: amoebiasis was enriched at high elevations, whereas atrazine degradation and siderophore synthesis were more active at low elevations. In high‐elevation habitats, glycan biosynthesis and siderophore synthesis were downregulated, as were pathways for pesticide degradation and bacterial epithelial cell invasion. In high‐precipitation habitats, pesticide degradation, siderophore synthesis, and bacterial infectious disease pathways were enriched. In high‐temperature‐seasonality habitats, pathways for development, macrolide synthesis, and arachidonic acid metabolism were enriched.

### 3.4 Modulatory Effects of Sex on the Gut Microbiota Community Structure

Comparisons of alpha diversity between sexes showed no significant differences in Chao1 or Simpson indices. PERMANOVA on Bray‐Curtis distances also revealed no significant effect of sex, and PCoA showed interdigitated distributions without sex‐specific clusters.

**FIGURE 5:**
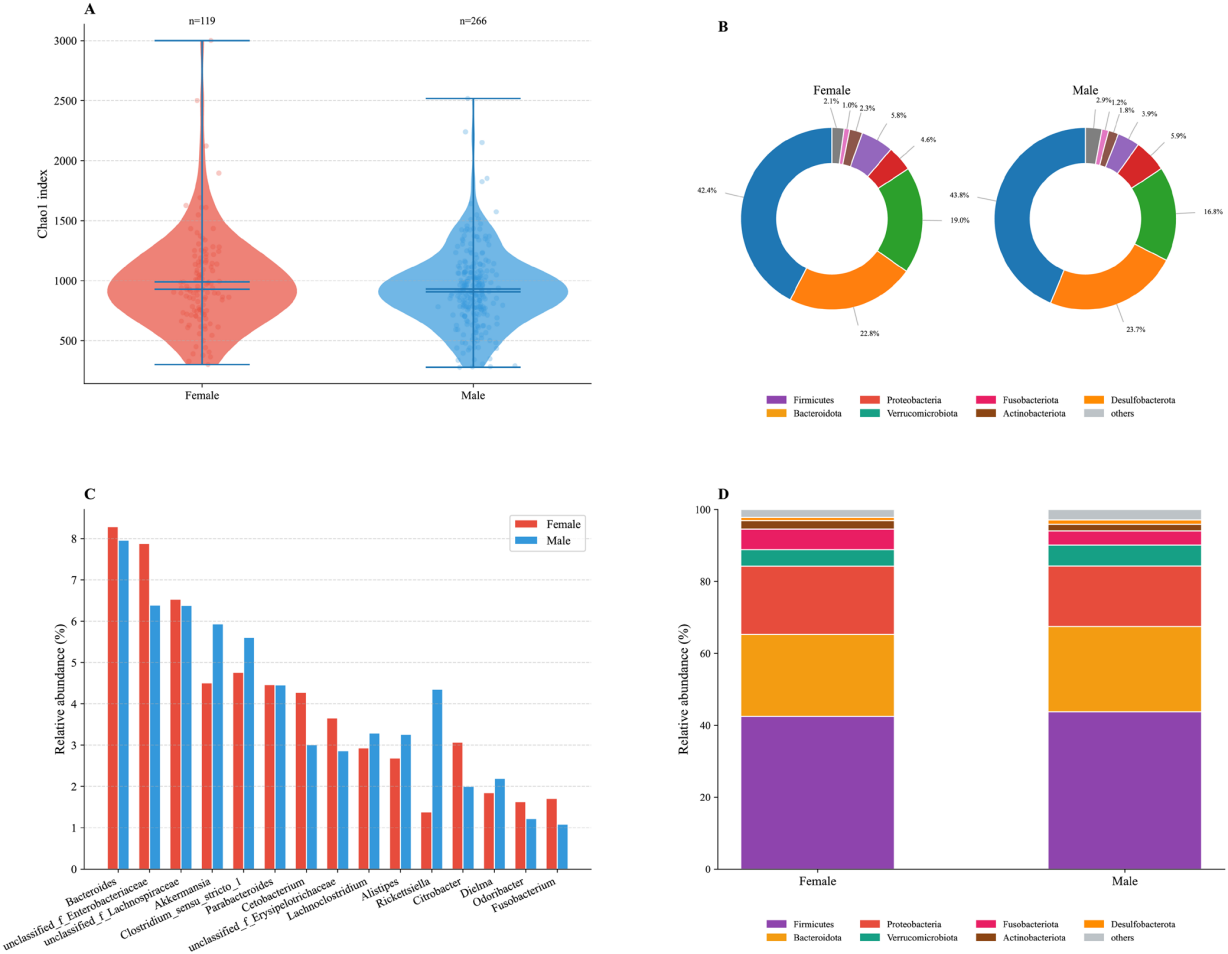
Sex effects on the gut microbiota of *D. melanostictus*. (A) Violin plots showing the distribution of Chao1 index between female (*n* = 119) and male (*n* = 266) individuals. No significant difference was observed in α‐diversity between sexes. (B) Donut charts displaying the relative abundance of major bacterial phyla in female and male toads. (C) Grouped bar chart showing the relative abundance of differentially abundant genera between sexes. (D) Vertical stacked bar chart illustrating the phylum‐level community composition in female and male individuals.

At the phylum level, Fusobacteria and the genus Cetobacterium were significantly more abundant in females, whereas Verrucomicrobiota and the genus Akkermansia were more abundant in males (Figure 5A–D). At the genus level, Enterobacteriaceae_unclassified and Citrobacter were more abundant in females, while Rickettsiella and Clostridium sensu stricto 1 were more abundant in males.

### 3.5 Geographical Variation Patterns of the Gut Microbiota

**FIGURE 6:**
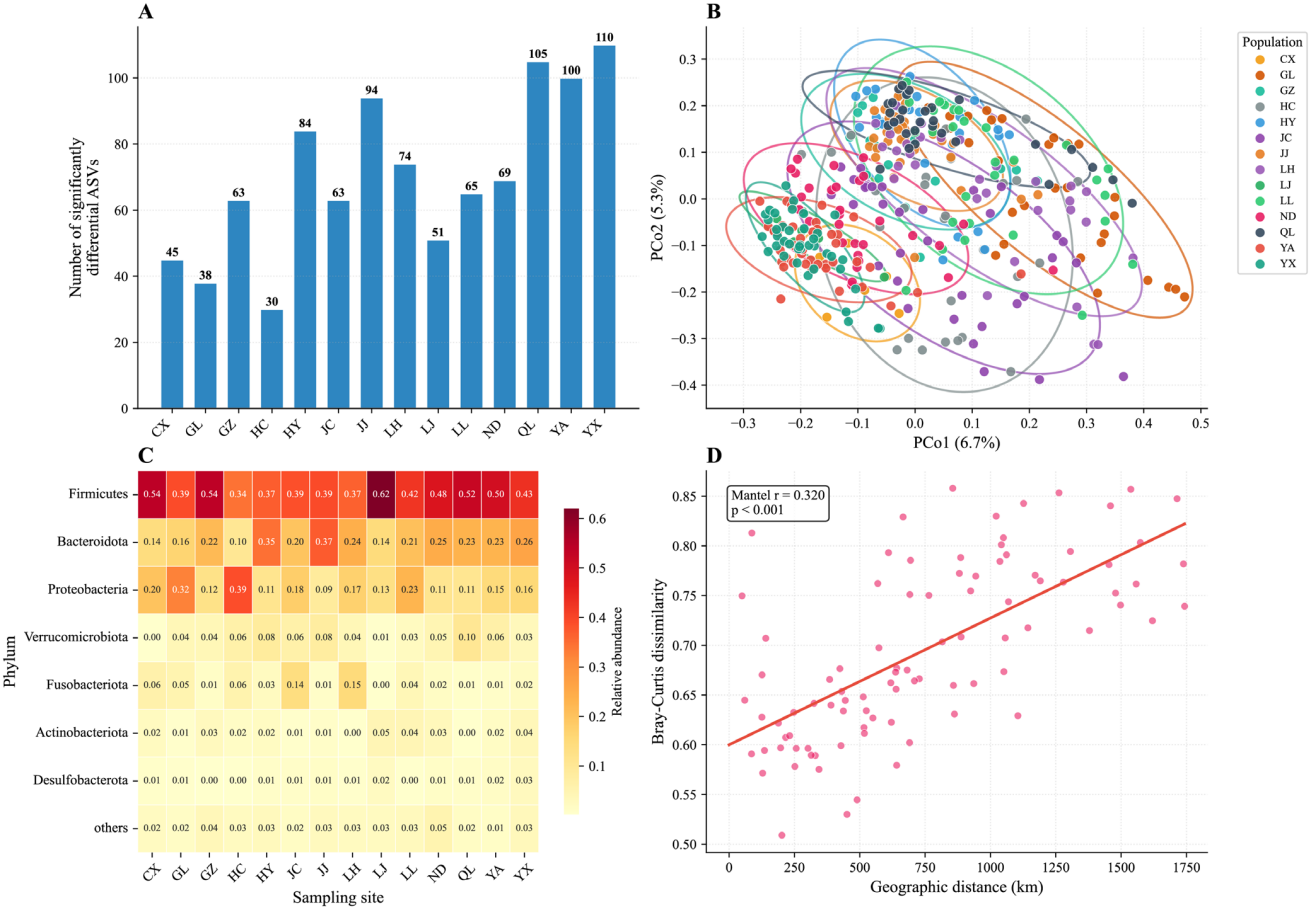
Geographical variation patterns of the gut microbiota. (A) Number of significantly differential ASVs per population (Kruskal–Wallis test, Dunn's post hoc, global *p* < 0.05). (B) Principal coordinate analysis (PCoA) of gut microbial communities based on Bray‐Curtis distances. Ellipses indicate 95% confidence intervals per population; variance explained is shown in parentheses. (C) Heatmap of phylum‐level relative abundances across 14 sampling sites. (D) Distance‐decay relationship between geographic distance (km) and Bray–Curtis dissimilarity among population pairs (Mantel test, *r* = 0.320, *p* < 0.001).

PCoA of the ASV relative abundance matrix showed that the first three principal components accounted for 15.93% of cumulative variance (Figure 6B). Community differences among populations were multidimensional and lacked strict geographic clustering, although populations with similar environments tended to cluster. The HC population was enriched in Proteobacteria (38.9%). The LH population was enriched in Fusobacteria (14.8%). The QL population showed peak Verrucomicrobiota abundance (10.3%). Mantel test confirmed a significant positive correlation between community dissimilarity and geographic distance (*r* = 0.319, *p* < 0.001; Figure 6A).

Along the latitudinal gradient, Firmicutes were enriched in lowlatitude populations (23.30° N–24.33° N, abundance 38.7%–61.9%), with the highest abundance in LJ (61.9%). In high‐latitude populations (26.19° N–27.12° N), Bacteroidota peaked in HY (34.6%), Verrucomicrobiota peaked in QL (10.3%), and Fusobacteria peaked in LH (14.8%). Along the altitudinal gradient, Firmicutes abundance in high‐elevation populations (1629–1911 m) ranged from 43.3% to 61.9% (LJ). Verrucomicrobiota and Bacteroidota were enriched at high elevations (YA: 6.5% and 23.0%, respectively). Fusobacteria were reduced at high elevations (YA: 1.0% vs. LH at low elevation: 14.8%). Along the longitudinal gradient, Firmicutes were more abundant in western populations (101.26° E–102.52° E), with LJ as the extreme (101.29° E). Bacteroidota and Fusobacteria were enriched in eastern populations (JJ: Bacteroidota 37.3%; LH: Fusobacteria 14.8%).

The Firmicutes phylum contained 11 ASVs detected in all 14 populations, with a low coefficient of variation (18.4%), indicating a stable core framework (Figure 6C,D).

### 3.6 Quantitative Analysis and Mechanism Explanation of the Driving Factors for Geographic Variation

**FIGURE 7:**
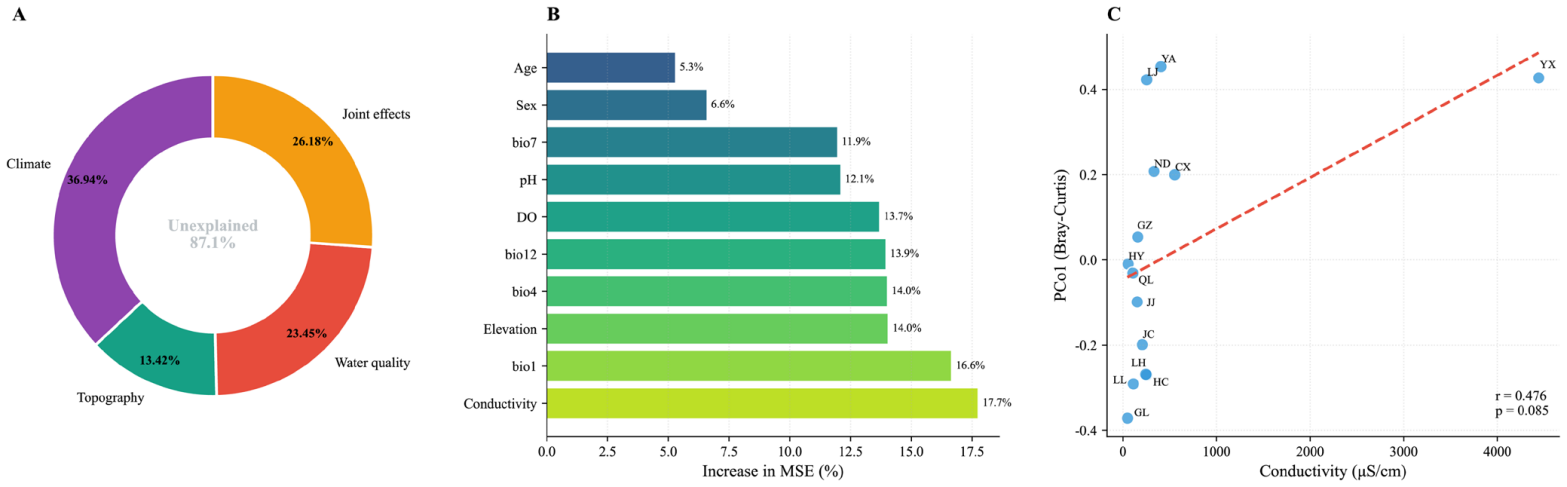
Driving factors of geographical variation in gut microbiota. (A) Variance partitioning of gut microbiota variation among climate, topography, water quality, joint effects, and unexplained fraction. (B) Random forest variable importance (%IncMSE) with conductivity, annual mean temperature, and elevation as the most influential factors. (C) Regression of PCoA axis 1 against water conductivity (*r* = 0.329, *p* < 0.001).

Variance partitioning analysis (VPA) showed that climate, topography, and water quality together explained 12.9% of the total variation (adjusted *R* ^2^ = 0.129, Figure 7A). Among the explained fraction, climate factors (bio1, bio4, bio7, bio12) contributed 4.77% (36.9% of explained), water quality (pH, conductivity, DO) contributed 3.03% (23.5% of explained), and topography (elevation) contributed 1.73% (13.4% of explained). Joint effects among the three groups accounted for approximately 3.4%, and 87.1% remained unexplained. Permanova explained 18.73% of total variation (*R* ^2^ = 0.187, *F* = 5.668, *p* = 0.001).

Random forest analysis (Figure 7B) ranked variable importance by %IncMSE: conductivity (17.7%), annual mean temperature bio1 (16.6%), elevation (14.0%), temperature seasonality bio4 (14.0%), annual precipitation bio12 (13.9%), DO (13.7%), pH (12.1%), bio7 (11.9%), sex (6.6%), and age (5.3%). Single‐factor importance assessment identified conductivity, mean annual temperature, and elevation as the three most influential variables (Figure 7C).

Climate PCA showed that PC1 (56.9%) and PC2 (25.3%) together explained 82.2% of climatic variation. Mantel test confirmed a distance‐decay relationship: Bray–Curtis dissimilarity increased with geographic distance (Mantel *r* = 0.320, *p* < 0.001).

## 4 Discussion

Variance decomposition analysis revealed that climate, topography, and water quality together explained 12.9% of the total variation (adjusted *R* ^2^ = 0.129), while host age and sex contributed only marginally. This supports an environment‐dominated model, but also raises the question of what drives the remaining approximately 87% of variation.

In microbial ecology, a large unexplained fraction is common, due to stochastic processes (e.g., dispersal limitation, ecological drift), unmeasured host factors (genetics, diet, behavior), and neutral dynamics. For host‐associated microbiomes, environmental factors frequently dominate over host genetics (Rothschild et al. 2018), and in amphibians, life stage and local habitat strongly shape gut microbiota, often overriding host genetic background (Song et al. 2021; Yang et al. 2022). Studies in other ecosystems—from estuarine sediments to terrestrial soils—have likewise shown that deterministic and stochastic processes operate in tandem, with environmental variables explaining only part of the variation (Chen, Lv, et al. 2023; Chen, Xu, et al. 2023; Riddley et al. 2025). Thus, the moderate explanatory power in this study does not diminish the biological importance of environmental filtering; it reflects the inherent complexity of gut microbiota assembly, where deterministic and stochastic forces act in concert.

Conductivity (a proxy for water salinity/ion content) was identified as the strongest predictor, followed by annual mean temperature and elevation. Elevation imposes screening pressure through vertical temperature gradients, and climate seasonality impacts community assembly via periodic fluctuations. Cross‐elevation studies on red‐backed salamanders similarly identified soil pH and substrate temperature as key factors shaping skin microbiota (Muletz Wolz et al. 2018), and bar‐headed geese studies showed that seasonal fluctuations have stronger screening effects than annual averages (Wang et al. 2017).

Although age does not alter overall geographic patterns, it can refine local communities through immune senescence. Actinobacteriota increased 23‐fold from ages 2–6, consistent with age‐related immune remodeling described in mammals (Bosco and Noti 2021; Pallikkuth et al. 2021; He et al. 2025). Sex effects were weak and group‐specific: Fusobacterium and Cetobacterium were more abundant in females, while Akkermansia and Rickettsiella were more abundant in males, aligning with studies on Asian toads (Song et al. 2021; Liu et al. 2024).

Microbial communities adapt to environmental stress via functional pathways. In seasonally variable climates, pathways for stress resistance (e.g., atrazine degradation, siderophore synthesis) were enriched, mirroring adaptation patterns in Svalbard reindeer (Sundset et al. 2009). Limitations of this study include the use of 16S rRNA sequencing (no strain‐level resolution), a cross‐sectional design, and spring‐biased sampling. Future work should use metagenomics, transplantation experiments, and temporal sampling to validate causal relationships.

The environment‐dominated pattern observed in *D. melanostictus* may apply to other widely distributed ectotherms with similar physiology, but caution is needed for narrow‐range endemic species or those with specialized diets, where host genetics or life history may play larger roles (Muletz Wolz et al. 2018; Sriswasdi et al. 2017). In conclusion, this study of 385 individuals from 14 populations demonstrates that water conductivity, climate, and elevation are the primary drivers of geographic variation in gut microbiota, while host age and sex have minor effects. These findings provide a quantitative framework for understanding amphibian gut microbiota adaptation and for predicting microbiome responses to environmental change.

## Author Contributions

**Di K. Zhu:** conceptualization (equal), data curation (lead), formal analysis (equal), writing – original draft (lead). **Nan Wen:** data curation (supporting), formal analysis (supporting). **Ke Y. Peng:** formal analysis (supporting), investigation (supporting). **Lin L. Jiang:** investigation (supporting), methodology (equal). **Jin Li:** conceptualization (equal), data curation (equal), formal analysis (lead), project administration (equal). **Li Zhao:** conceptualization (lead), data curation (equal), formal analysis (equal), funding acquisition (lead), investigation (lead), project administration (lead), resources (lead).

## Funding

This work was supported by the Doctoral Research Initiation Fund of China West Normal University, 21E036. College Students’ Innovation and Entrepreneurship Training Program of China West Normal University, 202310638059, 202410638117. National Natural Science Foundation of China, 32300358.

## Disclosure

Map description: The base map is downloaded from the standard map service of the Ministry of Natural Resources, China (GS(2024)0650), and no modifications have been made to the national boundaries.

## Conflicts of Interest

The authors declare no conflicts of interest.

## Supporting information


**Data S1:** ece373993‐sup‐0001‐DataS1.docx.

**Data S2:** ece373993‐sup‐0002‐DataS2.xlsx.

**Data S3:** ece373993‐sup‐0003‐DataS3.xlsx.

**Data S4:** ece373993‐sup‐0004‐DataS4.xlsx.

**Data S5:** ece373993‐sup‐0005‐DataS5.xlsx.

**Data S6:** ece373993‐sup‐0006‐DataS6.xlsx.

**Data S7:** ece373993‐sup‐0007‐DataS7.xlsx.

## Data Availability

The raw 16S rRNA gene sequencing data generated in this study are available in the NCBI SRA database under BioProject PRJNA1436778 (https://www.ncbi.nlm.nih.gov/sra/PRJNA1436778). The filtered OTU table used for all statistical analyses and figure generation has been deposited in Figshare and is publicly available at https://doi.org/10.6084/m9.figshare.32574063.
