# Supplementary material for: Geographical Variation in the Gut Microbiota of Duttaphrynus melanostictus : Relative Contributions of Environmental Filtering and Host Physiological Modulation
Source: Ecol Evol. 2026 Jul 9;16(7):e73993. doi: 10.1002/ece3.73993 (PMC13348828; doi:10.1002/ece3.73993)
Supplement: Supplementary file 1 — Data S1: ece373993‐sup‐0001‐DataS1.docx. [file ECE3-16-e73993-s005.docx]

# ============================================================

# Duttaphrynus melanostictus gut microbiota geographic variation

# Manual VPA calculation (permission issue fixed)

# ============================================================

# Set working directory to the folder containing all input files

# e.g., setwd("path/to/your/data/folder")

# Alternatively, place the script in the same folder and run without setwd()

library(readxl)

library(vegan)

library(randomForest)

# ---------- 1. Read OTU table ----------

cat("Reading OTU table...\n")

otu_raw <- read_excel("otu.xlsx", sheet = 1)

n_col <- ncol(otu_raw)

sample_cols <- 2:(n_col - 7)

otu_mat <- as.matrix(otu_raw[, sample_cols])

mode(otu_mat) <- "numeric"

otu_mat[is.na(otu_mat)] <- 0

rownames(otu_mat) <- otu_raw[[1]]

otu_t <- t(otu_mat)

total_abund <- colSums(otu_t)

presence <- colSums(otu_t > 0) / nrow(otu_t)

keep <- total_abund >= 10 & presence >= 0.05

otu_t <- otu_t[, keep]

cat("Samples:", nrow(otu_t), " ASVs:", ncol(otu_t), "\n")

# ---------- 2. Sex and age ----------

date_df <- read_excel("date.xlsx", sheet = 1)

date_df <- date_df[, c("Sample_Code", "sex", "years")]

names(date_df) <- c("SampleID", "Sex", "Age")

date_df$Age <- as.numeric(date_df$Age)

# ---------- 3. Environmental data ----------

env_site <- read_excel("env_data.xlsx", sheet = 1)

names(env_site)[names(env_site) == "Conductivity (μs/cm)"] <- "Conductivity"

names(env_site)[names(env_site) == "DO_mg/L"] <- "DO"

names(env_site)[names(env_site) == "ph"] <- "pH"

env_site$Site <- as.character(env_site$Site)

env_vars <- c("bio1","bio4","bio7","bio12","Elevation","pH","Conductivity","DO","Longitude","Latitude")

for(v in env_vars) env_site[[v]] <- as.numeric(env_site[[v]])

# ---------- 4. Merge metadata ----------

samples <- rownames(otu_t)

site_code <- substr(samples, 1, 2)

metadata <- data.frame(SampleID = samples, Site = site_code, stringsAsFactors = FALSE)

metadata <- merge(metadata, date_df, by = "SampleID", all.x = TRUE)

metadata <- merge(metadata, env_site, by = "Site", all.x = TRUE)

rownames(metadata) <- metadata$SampleID

metadata <- metadata[samples, ]

# Fix JC01 (if missing values)

if("JC01" %in% metadata$SampleID){

idx <- which(metadata$SampleID == "JC01")

if(is.na(metadata$Age[idx])) metadata$Age[idx] <- 5

if(is.na(metadata$Sex[idx])) metadata$Sex[idx] <- "female"

jc_env <- env_site[env_site$Site == "JC", ]

if(nrow(jc_env) == 1){

for(v in env_vars){

if(is.na(metadata[idx, v])) metadata[idx, v] <- jc_env[[v]]

}

}

}

complete <- complete.cases(metadata[, c("Age","Sex",env_vars)])

otu_t_clean <- otu_t[complete, ]

metadata_clean <- metadata[complete, ]

cat("Final sample size:", nrow(metadata_clean), "\n")

# ---------- 5. Shannon diversity ----------

shannon <- diversity(otu_t_clean, index = "shannon")

metadata_clean$Shannon <- shannon

# ---------- 6. Bray-Curtis distance ----------

dist_bc <- vegdist(otu_t_clean, method = "bray")

# ---------- 7. PERMANOVA ----------

perm_data <- metadata_clean[, c("Age","Sex","Site","pH","Conductivity","DO","Elevation","bio1")]

perm_data$Sex <- as.factor(perm_data$Sex)

perm_data$Site <- as.factor(perm_data$Site)

set.seed(123)

permanova <- adonis2(dist_bc ~ Age + Sex + Site + pH + Conductivity + DO + Elevation + bio1,

data = perm_data, permutations = 999)

write.csv(as.data.frame(permanova), "R_results/permanova_results.csv", row.names = TRUE)

# ---------- 8. Mantel test ----------

coords <- metadata_clean[, c("Longitude","Latitude")]

dist_geo <- dist(coords, method = "euclidean")

set.seed(123)

mantel_test <- mantel(dist_bc, dist_geo, method = "pearson", permutations = 9999)

mantel_out <- data.frame(r = mantel_test$statistic, p = mantel_test$signif)

write.csv(mantel_out, "R_results/mantel_result.csv", row.names = FALSE)

# ---------- 9. Manual variance partitioning ----------

otu_hell <- decostand(otu_t_clean, method = "hellinger")

clim <- scale(metadata_clean[, c("bio1","bio4","bio7","bio12")])

topo <- scale(metadata_clean[, "Elevation", drop = FALSE])

water <- scale(metadata_clean[, c("pH","Conductivity","DO")])

all_vars <- cbind(clim, topo, water)

colnames(all_vars) <- c("bio1","bio4","bio7","bio12","Elevation","pH","Conductivity","DO")

all_vars <- as.data.frame(all_vars)

complete <- complete.cases(all_vars)

otu_hell <- otu_hell[complete, ]

all_vars <- all_vars[complete, ]

rda_full <- rda(otu_hell ~ bio1 + bio4 + bio7 + bio12 + Elevation + pH + Conductivity + DO, data = all_vars)

adj_full <- RsquareAdj(rda_full)$adj.r.squared

rda_no_clim <- rda(otu_hell ~ Elevation + pH + Conductivity + DO, data = all_vars)

adj_no_clim <- RsquareAdj(rda_no_clim)$adj.r.squared

net_clim <- adj_full - adj_no_clim

rda_no_topo <- rda(otu_hell ~ bio1 + bio4 + bio7 + bio12 + pH + Conductivity + DO, data = all_vars)

adj_no_topo <- RsquareAdj(rda_no_topo)$adj.r.squared

net_topo <- adj_full - adj_no_topo

rda_no_water <- rda(otu_hell ~ bio1 + bio4 + bio7 + bio12 + Elevation, data = all_vars)

adj_no_water <- RsquareAdj(rda_no_water)$adj.r.squared

net_water <- adj_full - adj_no_water

unexplained <- 1 - adj_full

vpa_summary <- data.frame(

Component = c("Climate (net)", "Topography (net)", "Water quality (net)", "Unexplained"),

Adj_R_squared = c(net_clim, net_topo, net_water, unexplained)

)

cat("\n=== Variance Partitioning Results ===\n")

print(vpa_summary)

if(!dir.exists("R_results")) dir.create("R_results")

out_file <- "R_results/variance_partitioning_summary.csv"

if(file.exists(out_file)) file.remove(out_file)

write.csv(vpa_summary, out_file, row.names = FALSE)

# ---------- 10. Random forest ----------

rf_data <- metadata_clean[, c("Shannon","Age","Sex","bio1","bio4","bio7","bio12",

"Elevation","pH","Conductivity","DO")]

rf_data$Sex <- as.factor(rf_data$Sex)

rf_data <- na.omit(rf_data)

set.seed(456)

rf_model <- randomForest(Shannon ~ ., data = rf_data, ntree = 500, mtry = 3, importance = TRUE)

rf_imp <- data.frame(IncMSE = importance(rf_model, type = 1)[,1])

rf_imp$Variable <- rownames(rf_imp)

rf_imp <- rf_imp[order(-rf_imp$IncMSE), ]

write.csv(rf_imp, "R_results/random_forest_importance.csv", row.names = FALSE)

# ---------- 11. RDA ----------

rda_env <- metadata_clean[, c("bio1","bio4","bio7","bio12","Elevation","pH","Conductivity","DO")]

rda_result <- rda(otu_hell ~ ., data = rda_env, scale = TRUE)

site_scores <- as.data.frame(scores(rda_result, display = "sites", choices = 1:2))

site_scores$Site <- metadata_clean$Site

site_scores$SampleID <- rownames(metadata_clean)

env_scores <- as.data.frame(scores(rda_result, display = "bp", choices = 1:2))

env_scores$Factor <- rownames(env_scores)

write.csv(site_scores, "R_results/rda_site_scores.csv", row.names = FALSE)

write.csv(env_scores, "R_results/rda_environment_scores.csv", row.names = FALSE)

cat("\nAll analyses completed. Results saved in R_results folder.\n")
